# Gender-based comparison of clinical, epidemiological, and laboratory characteristics in hemodialysis patients: a retrospective analysis

**DOI:** 10.1590/1806-9282.20250982

**Published:** 2026-03-30

**Authors:** Alexandre Vizzuso de Oliveira, Júlia Ferreira Rocha, Beatrice Borges Sato, Rafaela Francisquetti Barnes, Caio Oliveira Bastos, Jonatas Lourival Zanoveli Cunha, Vívian Cristine Lima de Almeida, Géssika Marcelo Gomes, Gabriel Teixeira Montezuma Sales, Érika Bevilaqua Rangel

**Affiliations:** 1 Universidade Federal de São Paulo, Escola Paulista de Medicina – São Paulo (SP), Brazil.; 2 Universidade Federal de São Paulo, Escola Paulista de Medicina, Department of Medicine, Division of Nephrology – São Paulo (SP), Brazil.

**Keywords:** Diagnostic tests, routine, Gender, Hemodialysis

## Abstract

**OBJECTIVE:**

The aim of this study was to analyze and compare the clinical, epidemiological, and laboratory characteristics of male and female patients with kidney disease admitted for hemodialysis.

**METHODS:**

This retrospective, observational, cross-sectional study analyzed the clinical, epidemiological, and laboratory data from electronic health records of patients admitted for hemodialysis at a university hospital between January 2022 and November 2023, comparing men’s and women’s profiles. All adult patients were consecutively included, except those with missing data.

**RESULTS:**

A total of 163 patients were included: 53.9% of whom were male and 43.4% self-identified as white. Chronic kidney disease was diagnosed at admission in 78.5% of cases, with diabetes mellitus being the leading etiology, affecting 20.4% of men and 26.6% of women. Men had significantly higher serum creatinine (7.9 vs. 6.3 mg/dL; p<0.001), potassium (4.8 vs. 4.6 mEq/L, p=0.039), and phosphorus levels (5.9 vs. 5.0 mg/dL; p=0.004), whereas women had significantly higher high-density lipoprotein cholesterol cholesterol (46 vs. 37 mg/dL; p<0.001), low-density lipoprotein cholesterol cholesterol (120.4 vs. 97.8 mg/dL; p=0.004), and total cholesterol (198 vs. 161 mg/dL; p<0.001). Among patients with diabetes mellitus, women more frequently used statins (67.7 vs. 42.4%; p=0.042) and angiotensin II type 1 receptor blockers (58.1 vs. 27.3%; p=0.013). They also had higher total cholesterol (199 vs. 156 mg/dL; p=0.005) and low-density lipoprotein cholesterol cholesterol levels (117 vs. 92 mg/dL; p=0.043), while men had higher serum phosphorous levels (6.3 vs. 5.0 mg/dL; p=0.028).

**CONCLUSION:**

Significant gender-based differences were identified in the clinical, epidemiological, and laboratory characteristics of patients admitted for hemodialysis. Men presented higher serum creatinine, phosphorus, and potassium levels, whereas women showed higher cholesterol fractions and greater use of statins and angiotensin II type 1 receptor blockers.

## INTRODUCTION

Despite the availability of various renal replacement therapy (RRT) modalities, approximately 88% of the 157,000 Brazilians undergoing dialysis in 2023 were receiving conventional hemodialysis (HD)^1^. The majority of these patients reside in the Southeast region, which accounts for nearly 60% of the national HD population^2^. In São Paulo, the prevalence of patients on HD reaches 57.7 cases per 100,000 inhabitants, reflecting the high regional demand for this treatment.

Indications for HD may arise from either acute or chronic kidney conditions. Acute kidney injury (AKI) is defined as a sudden decline in renal function. Its management remains challenging, particularly regarding the timely reversal of renal insult and prevention of progression to chronic kidney disease (CKD). Depending on the clinical scenario, RRT—most commonly HD—may be required^3^.

CKD is characterized by a gradual and irreversible decline in kidney function, ultimately requiring RRT in advanced stages^4^. In Brazil, the leading causes of CKD are systemic arterial hypertension (SAH) and diabetes mellitus (DM)^1^. Globally, CKD affects an estimated 9.1% of the population (8.5–9.8%)^5^. Among individuals with DM, 20–40% develop CKD and are at increased risk for cardiovascular complications^5,6^. Cardiovascular disease (CVD) and kidney dysfunction are closely linked^5,6^.

Management strategies for CKD and CVD include the use of angiotensin-converting enzyme (ACE) inhibitors and angiotensin II receptor blockers (ARBs) for reno- and cardioprotection across all disease stages. In recent years, sodium-glucose cotransporter 2 inhibitors (SGLT2i), mineralocorticoid receptor antagonists, and GLP-1 receptor agonists have also demonstrated significant benefits in both renal and cardiovascular outcomes^6,7^.

Additionally, gender-based differences in CKD and CVD prevalence and in metabolic and cardiovascular outcomes have been widely discussed in the literature^8^.

Given this context, the present study aimed to analyze and compare the clinical, epidemiological, and laboratory profiles of male and female patients with kidney disease who were admitted for hemodialysis at Hospital São Paulo (HSP), a leading university referral center, based on real-world data.

## METHODS

The study was approved by the Research Ethics Committee of the Federal University of São Paulo (CAAE 7954624.0.0000.5505; October 4, 2024). The Committee formally waived the requirement for written informed consent, as most patients had been transferred to other HD centers, making it difficult to contact them despite reasonable efforts. Furthermore, the Committee determined that the study involved minimal risk, that all clinical data and images were fully anonymized, and that the waiver would not adversely affect the rights or welfare of the patients.

This single-center, observational, retrospective, cross-sectional study included patients admitted to the hemodialysis unit of HSP, a public university referral center for dialysis, between January 2022 and November 2023. All patients with CKD, acute-on-CKD, or AKI were consecutively included. Data were obtained from electronic health records.

Inclusion criteria were: admission for HD and availability of complete admission forms and medical records in the HSPaffiliated systems. Patients with partially or completely missing data were excluded. Of 193 medical records reviewed, 30 were excluded for not meeting the eligibility criteria.

Clinical–epidemiological data, including gender, age, ethnicity, CKD etiology, type and number of comorbidities, and chronic medications (e.g., insulin, biguanides, sulfonylureas, erythropoietin, vitamin D, diuretics, calcium channel blockers, beta-blockers, ACEi, ARBs, and SGLT2i), were extracted from the HD unit admission forms. Laboratory parameters were retrieved from electronic health records. Estimated glomerular filtration rate (eGFR) was calculated using an equation incorporating age, gender, and serum creatinine levels.

Analyzed diseases included CKD, acute-on-CKD, AKI, SAH, DM, rheumatologic, hematologic, oncologic, cardiovascular, and cerebrovascular diseases. Lupus nephritis was classified separately from glomerular diseases.

Patients were stratified by gender to identify gender-based differences. The primary outcome was the analysis of clinical and laboratory parameters in patients admitted for HD. Secondary outcomes included clinical and laboratory differences among patients with diabetic kidney disease and the presence of prior cardiovascular or cerebrovascular events. These events were categorized as acute myocardial infarction (AMI) with or without ST-segment elevation, ischemic or hemorrhagic stroke, transient ischemic attack (TIA), and events of undetermined cause. Disease classification was based on the 10th edition of the International Classification of Diseases (ICD-10).

Statistical analyses were performed using SPSS Statistics Version 2021. Categorical variables were expressed as absolute and relative frequencies; quantitative variables were presented as mean and standard deviation. Pearson’s chi-square test was used for categorical variable comparisons. After applying the Shapiro-Wilk normality test, the independent t-test was used for numerical variables with a normal distribution, and the results were expressed as mean±standard deviation. For numerical variables without a normal distribution, the Mann-Whitney test was applied, and the results were expressed as median and interquartile range (Q1–Q3). A p<0.05 was considered statistically significant. Graphs were created using GraphPad Prism Version 8.0.1 (Dotmatics, Boston, MA, USA).

## RESULTS

A total of 163 medical records were analyzed for patients admitted to the HD unit between January 2022 and November 2023. Of these, 88 (53.9%) were men and 75 (46.0%) were women. The mean age was 52.5±17.6 years, with no significant difference between genders (Table 1).

**Table 1:** Clinical, epidemiological, and comorbidity profiles of patients undergoing hemodialysis, stratified by gender.

Variables	All (n=163)	Men (n=88)	Women (n=75)	p-value
Race				
White (n, %)	70 (43.4%)	35 (40.6%)	35 (46.6%)	
Black (n, %)	23 (14.2%)	15 (17.4%)	8 (10.6%)	
Brown (n, %)	62 (38.5%)	33 (38.3%)	29 (38.6%)	
Asian (n, %)	6 (3.7%)	3 (3.4%)	3 (4.0%)	
Number of comorbidities (n)	3.9±2.2	4.0±2.3	3.9±2.1	0.414
Residual diuresis (mL)	906.0±708	925.0±788.6	883.0±605.5	0.753
Chronic kidney disease (n, %)	128 (78.5%)	69 (78.4%)	59 (78.6%)	0.968
Hypertension (n, %)	124 (76%)	66 (75%)	58 (77.3%)	0.728
Diabetes mellitus (n, %)	61 (37.4%)	30 (34.1%)	31 (41.3%)	0.341
Rheumatologic disease (n, %)	23 (14.1%)	11 (12.5%)	12 (16.0%)	0.542
Hematologic disease (n, %)	6 (3.6%)	4 (4.5%)	2 (2.67%)	0.521
Oncological disease (n, %)	12 (7.3%)	6 (6.8%)	6 (8.0%)	0.773
Cardio- or cerebrovascular disease (n, %)	22 (13.5%)	14 (63.6%)	8 (36.4%)	0.275
**Medications (n, %)**	**All (n=163)**	**Men (n=88)**	**Women (n=75)**	**p-value**
Biguanide	9 (5.5%)	4 (4.5%)	5 (6.7%)	0.555
Sulfonylurea	9 (5.5%)	4 (4.5%)	5 (6.7%)	0.555
Insulin	38 (23.3%)	19 (21.6%)	19 (25.3%)	0.573
Erythropoietin	32 (19.6%)	18 (20.5%)	14 (14.7%)	0.775
Vitamin D	33 (20.2%)	18 (20.5%)	15 (20.0%)	0.943
Statin	63 (38.7%)	28 (31.8%)	35 (46.7%)	0.052
Non-furosemide diuretic	45 (27.6%)	27 (30.7%)	18 (24.0%)	0.342
Furosemide	80 (49.1%)	41 (46.6%)	39 (52.0%)	0.491
Calcium channel blocker	69 (42.6%)	37 (42.5%)	32 (42.7%)	0.986
Beta-blocker	74 (45.7%)	39 (44.8%)	35 (46.7%)	0.851
ACEi	21 (12.9%)	11 (12.5%)	10 (13.3%)	0.874
ARB	54 (33.1%)	25 (28.4%)	29 (38.7%)	0.166

ACEi: angiotensin-converting enzyme inhibitor; ARB: angiotensin II AT1 receptor blocker.

**Figure 1:**
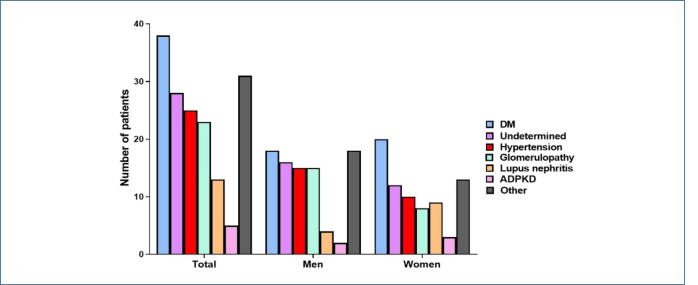
Etiologies of chronic kidney disease. DM: diabetes mellitus; ADPKD: autosomal dominant polycystic kidney disease.

At admission, 128 (78.5%) patients were diagnosed with CKD, while 21.5% presented with acute-on-CKD or AKI. In women, the main CKD etiologies were DM (26.6%), undetermined (16.0%), and SAH (13.3%). Among men, the most frequent causes were DM (20.4%), undetermined (18.1%), SAH (17%), and glomerulopathies (17%) (Figure 1).

Although SAH was not the leading cause of CKD, 76% of patients had hypertension as a comorbidity and 64 patients (39.2%) had DM. Other comorbidities included rheumatologic, hematologic, oncologic, and cardiovascular/cerebrovascular diseases. The number of comorbidities per patient was not different between men and women. Additionally, 31.9% had a history of smoking and 14.1% reported alcohol use. Eye disorders were observed in 23 (14.1%) patients: 52.1% with hypertensive or diabetic retinopathy, 26.0% with glaucoma, 17.3% with cataracts, and 4.3% with retinitis pigmentosa. Hypothyroidism was documented in 12.8% of patients. A history of cardiovascular or cerebrovascular events was present in 13.5% of patients, with 40.9% having AMI, 36.4% stroke, 18.2% TIA, and 4.5% undetermined events.

In terms of medication, 49.1% were using furosemide and 46% used ACEi or ARBs. Insulin was used by 23.3% of patients. Erythropoietin was already being used by 19.6% of patients for CKD-related anemia. Statin use was also common (38.7%), slightly more frequent among women, though not statistically significant (Table 1).

Serum creatinine levels were significantly higher in men, although no difference was seen in eGFR. Serum phosphorus was higher in men (p=0.002). Lipid profile analysis showed that women had significantly higher total cholesterol (p<0.001), HDL (p=0.004), and LDL (p=0.004) compared to men (Table 2).

**Table 2:** Laboratory parameters of male and female patients admitted for hemodialysis.

Laboratory tests	All (n=163)	Men (n=88)	Women (n=75)	p-value
Hemoglobin (g/dL)	9.0 [7.9; 10.5]	8.8 [7.8; 10.6]	9.1 [8.0; 10.3]	0.967
Serum creatinine (mg/dL)	7.0 [5.3; 9.5]	7.9 [6.1; 10.3]	6.3 [4.6; 8.5]	***<0.001***
eGFR (mL/min/1.73 m^2^)	7.4 [4.9; 9.9]	7.4 [5.5; 9.7]	7.7 [4.8; 10.3]	0.988
Urea (mg/dL)	163 [128; 214]	174 [133; 227]	160 [124; 211]	0.232
Sodium (mEq/L)	136 [133.2; 139]	136 [133.2; 139]	137 [133.5; 139]	0.466
Potassium (mEq/L)	4.7 [4.3; 5.4]	4.8 [4.4; 5.5]	4.6 [4.1; 5.1]	***0.039***
Fasting glucose (mg/dL)	100 [85; 124.5]	100 [86; 128]	107 [83.7; 121]	0.779
Glycated hemoglobin (%)	5.6 [5.1; 6.7]	5.9 [5.2; 6.9]	5.5 [4.8; 5.9]	0.753
Ionized calcium (mmol/L)	1.18 [1.13; 1.25]	1.18 [1.13; 1.25]	1.18 [1.12; 1.25]	0.753
Phosphorus (mg/dL)	5.3 [4.4; 6.5]	5.9 [4.9; 7.4]	5.0 [4.2; 5.9]	***0.004***
pH	7.3±0.0	7.3±0.0	7.3±0.0	0.219
Bicarbonate (mEq/L)	18.6±4.5	18.4±4.6	18.8±4.5	0.623
Total cholesterol (mg/dL)	178.2±50.2	161.4±46.1	198.0±47.8	***<0.001***
HDL (mg/dL)	40 [33; 53]	37 [31; 46]	46 [38; 55.5]	***<0.001***
LDL (mg/dL)	108.1±41.1	97.8±39.3	120.4±40.0	***0.004***
Triglycerides (mg/dL)	136.5 [92.2; 197.7]	130.5 [90; 188.7]	139 [95; 210]	0.304
Parathyroid hormone (pg/mL)	261.0 [112.1; 420]	214 [62.5; 378]	289 [168.2; 479.7]	0.221
Alkaline phosphatase (U/L)	96.5 [71.2; 133]	84.5 [66.2; 121.7]	114.5 [74.5; 136.2]	0.094

eGFR: estimated glomerular filtration rate; HDL: high-density lipoprotein cholesterol; LDL: low-density lipoprotein cholesterol. All variables are expressed as median and interquartile range [Q1; Q3], except for pH, bicarbonate, total cholesterol, and LDL, which are expressed as mean±standard deviation. The bold and italic values represent the significant p-values.

Given the high prevalence of DM, a subgroup analysis of diabetic patients showed a mean age of 60.9±12.2 years with no gender difference. Comorbidity count and residual diuresis were similar between genders. Among diabetic patients, 57.8% used insulin and 54.7% used statins, with greater statin use in women (67.7% vs. 42.4%; p=0.042). ARB use was significantly more common among diabetic women than men (58.1 vs. 27.3%; p=0.013). Additionally, among diabetic patients, men exhibited higher phosphorous levels (6.3 [3.9; 7.9] vs. 5.0 [4.2; 5.6] mg/dL; p=0.028), whereas women had higher total cholesterol (199.3±51.8 vs. 156.0±45.4 mg/dL; p=0.005), LDL (117.2±45.2 vs. 92.0±32.7 mg/dL; p=0.043), and HDL levels (51 [43.5; 59.5] vs. 36 [29.5; 54] mg/dL; p=0.016). Potassium concentrations were comparable between diabetic men and women (4.9 [4.4; 5.5] vs. 4.6 [4.2; 5.0] mEq/L; p=0.1), as were triglycerides (116 [90; 196] vs. 110 [78; 223] mg/dL; p=0.7).

## DISCUSSION

Analysis of the 163 patients initiating hemodialysis at HSP revealed a higher prevalence in men, with most individuals in their sixth decade of life. The primary etiology of CKD was DM, followed by undetermined causes and SAH. Laboratory abnormalities differed by gender: women had more pronounced dyslipidemia in both the general population and diabetic subgroups, while men showed higher serum phosphorus levels, including among diabetics.

Overall, the patient profile mirrored findings from other Brazilian regions according to the last census, with a predominance of older and white males in dialysis^1^. Laboratory alterations identified—particularly in hemoglobin, phosphorus, and parathyroid hormone levels—are common complications in CKD progression, including anemia, hyperphosphatemia, and secondary hyperparathyroidism^2,4,5^.

Approximately one-third of the cohort reported a history of smoking, a known modifiable risk factor for CKD progression^9^. Hypothyroidism, also frequently observed, is linked to declining renal function via multiple mechanisms, including glomerular basement membrane changes, reduced tubular mass, and diminished cardiac output^10^.

A considerable number of patients had prior cardiovascular or cerebrovascular dysfunction. CVD remains a leading cause of death among CKD patients, with ventricular hypertrophy, heart failure, and ischemic heart disease becoming more common as renal function declines^4,5^. Similarly, cerebrovascular events are associated with mineral bone disorder, uremic toxins, acid–base disturbances, and anemia—factors that increase stroke risk by over 40% from CKD stage 3 onward^11^.

In contrast to national data that list SAH as the most common CKD etiology^1^, SAH ranked third in our cohort. DM was the leading cause in both genders, aligning with international trends^7^. Although 76% of the patients had hypertension, it may often be secondary to CKD from another primary cause. The lack of fundoscopic exams limited further evaluation of hypertensive retinopathy.

Men had higher serum creatinine levels, which were expected due to greater muscle mass and metabolism differences^12^. This reinforces the limitations of isolated creatinine values, with eGFR offering a more accurate renal function assessment. Interestingly, men also presented with higher serum phosphorus levels. Although the literature often reports elevated phosphorus in postmenopausal women—potentially due to hormonal changes^13^—we did not assess menopausal status in our cohort. Evidence indicates that estrogen therapy in postmenopausal women is associated with lower serum phosphorus levels, likely through enhanced phosphaturia^14^. In addition, poor adherence to dietary phosphate restrictions and phosphate binder use, previously reported more frequently among men in other settings^8,15^, may contribute to our findings. Nevertheless, some studies suggest that gender-related adherence may actually favor men^16^.

Regarding lipid profiles, women exhibited significantly higher total cholesterol, HDL, and LDL levels—differences likely driven by hormonal and metabolic changes in postmenopausal women. Estrogen normally promotes LDL receptor expression, and its reduction leads to higher LDL and a more atherogenic profile^17^. While men maintain stable testosterone levels, their lipid metabolism is less affected by age.

Although women had higher HDL, this cardioprotective effect may be impaired in CKD due to structural and metabolic changes that reduce HDL functionality^18^. Despite more frequent statin use among women, concerns remain about underdosing or lower adherence, potentially due to adverse effects. Additionally, women are often underrepresented in statin trials^19^, raising questions about dosing and efficacy. Our finding that only 54.7% of diabetic patients used statins highlights the need to reinforce indications for statin therapy in CKD^6,7^.

Over half of the patients were on ACE inhibitors or ARBs. These agents are cornerstone therapies in CKD, particularly in diabetic populations, for renal and cardiovascular protection^6,7^. Greater ARB use among diabetic women may suggest better tolerance and lower discontinuation rates due to side effects such as hyperkalemia.

Non-diabetic men exhibited higher serum potassium levels than women, consistent with previous findings in men with non-dialysis CKD stages G3–G5, particularly among smokers^20^. Nevertheless, hyperkalemia affects nearly 50% of patients with CKD stages 4–5, regardless of gender^20^, and is closely linked to impaired renal function as well as the use of ACE inhibitors, ARBs, or potassium-sparing diuretics—factors that may contribute to cardiovascular events^21,22^.

Elevated potassium levels may also reflect dietary patterns or indicate more advanced diabetic nephropathy, potentially associated with hyporeninemic hypoaldosteronism^22^. Further investigation into treatment adherence and disease severity is warranted.

Notably, 5% of patients were still receiving metformin at the time of HD initiation, despite its contraindication in individuals with an eGFR <30 mL/min/1.73 m^2^ due to the risk of lactic acidosis^23^. This finding underscores the need for ongoing medical education to ensure the safe and appropriate use of medications in patients with advanced CKD.

This study has limitations: retrospective design has weaknesses, since the inclusion criteria were based on data collected for other purposes; were often not homogeneous; and had missing data. Its retrospective design also led to the exclusion of patients with incomplete records; not all patients had complete laboratory data; we could not fully evaluate patients with acute-on-CKD or AKI due to incomplete clinical evolution data; and adherence to medications, their doses, and dietary recommendations was not assessed.

## CONCLUSION

This study identified gender-based differences in the clinical and laboratory profiles of patients admitted for HD. Men presented higher serum creatinine, phosphorus, and potassium levels, whereas women had higher cholesterol fractions and reported greater use of statins and ARBs. These findings highlight distinct clinical and laboratory patterns between male and female patients admitted for HD.

## Data Availability

The datasets generated and/or analyzed during the current study are available from the corresponding author upon reasonable request.
